# Discrepancies between perceptions of students and deans regarding the consequences of restricting students’ use of electronic medical records on quality of medical education

**DOI:** 10.1186/s12909-017-0887-2

**Published:** 2017-03-13

**Authors:** Ivan Solarte, Karen D. Könings

**Affiliations:** 1 Pontificia Universidad Javeriana School of Medicine, Hospital Universitario San Ignacio, Carrera 7 40-62, Bogota, Colombia; 2 Department of Educational Development & Research and Graduate School of Health Professions Education, Maastricht University, Maastricht, The Netherlands

**Keywords:** Electronic medical records, Electronic health records, Undergraduate medical education, Clerkships, Workplace learning, e-learning, Student perceptions, Stakeholders

## Abstract

**Background:**

Electronic medical records (EMR) are more used in university hospitals, but the use of EMR by medical students at the workplace is still a challenge, because the conflict of interest between medical accountability for hospitals and quality of medical education programs for students. Therefore, this study investigates the use of EMR from the perspective of medical school deans and students, and determines their perceptions and concerns about consequences of restricted use of EMR by students on quality of education and patient care.

**Methods:**

We administered a large-scale survey about the existence of EMR, existing policies, students’ use for learning, and consequences on patient care to 42 deans and 789 Residency Physician Applicants in a private university in Colombia. Data from 26 deans and 442 former graduated students were compared with independent t tests and chi square tests.

**Results:**

Only half of medical schools had learning programs and policies about the use of EMR by students. Deans did not realize that students have less access to EMR than to paper-based MR. Perceptions of non-curricular learning opportunities how to write in (E)MR were significantly different between deans and students. Limiting students use of EMR has negative consequences on medical education, according to both deans and students, while deans worried significantly more about impact on patient care than students. Billing issues and liability aspects were their major concerns.

**Conclusions:**

There is a need for a clear policy and educational program on the use of EMR by students. Discrepancies between the planned curriculum by deans and the real clinical learning environment as experienced by students indicate suboptimal learning opportunities for students. Creating powerful workplace-learning experiences and resolving concerns on students use of EMR has to be resolved in a constructive collaboration way between the involved stakeholders, including also EMR designers and hospital administrators. We recommend intense supervision of students’ work in EMR to take full advantage of the technological advances of EMR at the modern clinical site, both for patient care and for medical education.

## Background

Information and communication technologies are an important part of today’s health systems, including university hospitals and therefore medical education. Worldwide, most hospitals are evolving towards the development of electronic medical records (EMR) including standardized entries, suggested prescription and online monitoring of guidelines adherence [1–3]. For medical students of the “net generation” it is more natural to write on a computer and look for information on the internet than to work with handwritten notes on a chart [4]. However, “medical students are learners. Thus, states do not give licenses to them and their notes should not become part of the medical-legal record” [5]. This has been identified by several educational associations such as the Association of American Medical Colleges (AAMC) and the Council of Medical Education (CME) that have issued reports calling for changes in undergraduate and graduate curricula to better educate students and residents in this critical aspect of medical practice [5–7]. The Alliance for Clinical Education states that students must document their own findings in EMR to develop written communication skills and clinical reasoning. The Alliance called for medical educators to have a unified policy in this regard [8]. Understanding of effects of the transition of paper-based medical records to EMR on medical education is still limited.

Medical students are unlicensed and therefore, they are not allowed to independently work in an EMR and report on examination or medical decision-making, but they do have to learn the skills to work with EMR before graduation [5]. A discrepancy arises between interests of medical education and patient care. In order to get insight in the impact of these conflicting interests in practice, this study investigates the perceptions of deans of medical schools, who are well aware of liability issues and regulations, and students, who have to develop their competencies at the workplace, regarding students’ use of EMR, its consequences and felt concerns.

Students will be cautious and awaiting guidance in managing EMR from their teachers, but many medical schools have not changed their curricula to introduce competencies in manage EMR [9]. There is a need to consider the presence of computers as a potential disruptive “third party” on the student-patient relationship and on team work at medical ward.Since the introduction of EMR, the availability of patient information online has changed the way patients’ clinical history is obtained and reviewed, which is likely to affect the development of patient-student relationships and oral communication skills [8, 10, 11]. Students no longer collect information from patients and families before the presentation of the information to the group during the clinical round; instead students and teachers both collect most information about the patient by reviewing EMR in advance. It has been shown that training is needed to learn how to effectively use EMR and identify patient safety issues [12]. Furthermore, the development of written communication skills can be hindered by EMR, as a good proportion of hospitals prohibit data entry by students [6]. The use of the tool to copy and paste information, as well as the presence of programmed inputs to history have diminished the quality of clinical history and hampered the development of clinical reasoning [13].

Academic medical centers with departments of Health Informatics have designed systems to enable better use of EMR by students, but this is not the case in most hospitals [14]. Only some medical schools have incorporated the topic of adequate use of information technology in their curriculum; training programs for these skills, which are of high importance for today’s physicians, are too often lacking [9, 15]. Therefore, exploring the perspective of deans — the academic authorities — regarding the use of EMR by medical students in comparison to the views of the students themselves will add to our understanding of the challenges medical education is currently facing. Several earlier reported studies have conducted interviews with deans, program directors, clerkship directors, residents or medical students getting their opinions. In general, no positive effects of the implementation and use of EMR on medical education were reported [13, 16–18]. A potential problem with these studies is that contrasts between the viewpoints of different stakeholders have not been studied in depth. When lacking insight in the different perspectives on the same issues, it is hard to find clues to improve the situation [19]. The research question to be addressed is: Are there differences between the perceptions of deans and students regarding the use of EMR by students, its value in medical education, and its consequences for quality of medical education and patient care?

## Methods

### Participants and procedure

We invited all 42 deans of medical schools, being members of the Colombian Association of Medical Schools, to voluntary and anonymously participate in the study and fill out an online survey in February 2013. Seventeen deans and nine program directors on behalf of the deans responded (total response rate 62%), after sending two reminders.

Additionally, we invited 789 medical doctors, who graduated from several universities in Colombia and were applying to a residency program at Pontificia Universidad Javeriana in 2012. Former graduates, who were medical students in the last 3 years and have been users of EMR as students and doctors, represent the experienced student perspective. They are called ‘students’ throughout this article. A paper-based survey was handed out to them just before the presentation of their medical knowledge exam. It started with a paragraph asking for voluntary participation and assuring anonymity. 726 out of 789 (92%) completed the survey. We selected data from graduates in the last 3 years for current analyses, as they had recent experiences with EMR at medical school (*N* = 442, 49.4% female, 86% younger than 26 years old).

### Instrument

An existing questionnaire about the use of medical records (MR) and EMR in medical education [17] was used with the proper permission, translated into spanish and modified to our study context. We used two parallel versions — one for students and another for deans — containing 19 items,with small variations in the formulations depending of the targeted group. Items about the intended and actual student’use of MR and EMR to write progress notes were answered on a 4-point Likert type scale (from 1 = no hospitals to 4 = all hospitals). Questions about perception on the impact of students’ use of MR on education and patient care were written using the following general wording, for example: “What impact, if any, do you feel there is on medical student education if student notes are not allowed to be placed in patient records?” Questions were answered on a 5-point Likert scale (from 1 = very negative to 5 = very positive). Additionally, some items were included about demographic factors and characteristics of their medical schools: Number of students, number and type of affiliated hospitals, and if the school was private or public.

### Data analyses

Data were analyzed using SPSS 19 software. Data from deans and students were compared with independent *t*-tests and *χ* ^*2*^-tests. Data on characteristics of medical schools showed that the sample of students did not differ from the sample of deans in the percentage of public versus private schools, type of teaching hospitals, and number of affiliated hospitals (χ^2^ tests n.s.). To indicate effect sizes, Cohen’s *d* is reported: Cohen’s *d* of 0.2–0.3 is considered as small effect, around 0.5 as medium effect and larger than 0.8 as large effect [20].

## Results

### Perceptions of the use of electronic medical records

Regarding student access to use EMR, the score of students (*M* = 1.98, *SD* = .71) was significantly higher that the deans’ score (*M* = 1.57, *SD* = .60; *t* = -2.62, *p* < .01, *d* = 0.58). This makes evident a greater use of EMR by students at the affiliated hospitals than what was planned or expected by the deans. Table 1 summarizes the answers of two questions about all affiliated hospitals permission for students to write notes in paper or electronic MR per year of enrollment.

**Table 1:** Comparison of perceptions between students and deans regarding permission for students to place notes on paper MR or EMR

Year of enrollment and type of MR	Students		Deans				
	*M*	*SD*	*M*	*SD*	*T*	*p*	*d*
Year 3, paper	2.65	1.11	1.73	.88	−3.11	< .01	0.84
Year 3, EMR	2.40	1.16	1.53	.74	−2.86	< .01	0.77
Year 4, paper	2.96	.91	2.00	.88	−3.39	< .01	1.06
Year 4, EMR	2.75	1.04	1.83	.92	−3.59	< .01	0.89
Year 5, paper	3.19	.85	2.00	1.00	−5.47	< .01	1.38
Year 5, EMR	3.00	.95	1.89	.94	−4.86	< .01	1.17
Internship, paper	3.61	.75	2.70	1.13	−5.01	< .01	1.16
Internship, EMR	3.56	0.76	2.18	1.18	−4.16	< .01	0.92

Answered on a 4-point Likert type scale (from 1 = no hospitals to 4 = all hospitals)

Students reported that they had access to write notes in patient records in more hospitals than the deans believed (significant on all tests). Students perceived that more hospitals using paper-based MR, allowed them to place notes compared to hospitals using EMR, particularly during years 3 (*t* = 2.62, *p* = .02, *d* = 0.22) and 4 (*t* = 2.64, *p* = .01, *d* = 0.21). This difference in the hospital permission, which dependes on the kind of medical record, was not found in the deans’ perceptions.

### Educational program and learning opportunities on MR

The existence of a formal educational program on how to write progress notes in MR was recognized by 58.50% of the students and 57.50% of deans (*χ2* = .07, *p* = .97). Both students and deans perceived the program about writing and assessing progress notes was mainly taught in 3rd and 4th year (*χ2* = 3.44, *p* = .63 for write and *χ2* = 5.49, *p* = .36 for assess). About half of the students (54%) and the deans (46%) answered that there was a university policy that regulates the placement of medical students’ notes in patient medical records.

Students can also become skilled in writing progress notes from “non-curricular” learning opportunities. Table 2 shows that observing residents and physicians were most mentioned opportunities. However, deans considered ‘observing attending physicians’ to be a more important resource at the clinical site than students did (*p* = .01). Deans also considered “writing without permission” and “need to document in MR during the internship”, as more important learning opportunities to learn to write in MR than students did (*p* respectively .02 and .01).

**Table 2:** Non-curricular opportunities to learn how to write progress notes in MR, as indicated by students and deans

	Students %		Deans %		*χ* ^*2*^	*p*
	Yes	No	Yes	No		
Observing residents	27	73	31	69	0.16	.66
Observing attending physicians	31	69	58	42	8.14	.01
Writing without permission	3	97	15	85	9.07	.02
Need to document in MR during internship	10	90	27	63	24.07	.01

More than one answer was allowed

### Consequences of EMR use for education and patient care

Deans and students thought that students’ notes should be part of the EMR in inpatient services in a progressive way from 3^rd^ year (46% of students and 60% of deans, *χ* ^*2*^ = 3.40, *p* = .18) to 5^th^ year (85% of students and 96% of deans, *χ* ^*2*^ = 2.07 *p* = .36). Numbers were similar when they were asked about outpatient services.

Answers about the impact of restrictions for students to write in EMR on the quality of medical education showed that such restriction has negative impact, both according to students (*M* = 1.80; *SD* = .94) and for deans (*M* = 1.54, *SD* = .76, *t* = 1.66, *p* = .11, *d* = 0.28). When asked about specific consequences of the restriction of students’ writing in EMR, deans and students perceived negative consequences on five aspects of medical education: Student involvement in patient care, assessment of competences, feeling part of the team, development of communication written skills, and groundwork to be a MD (see Table 3). They did not differ in their opinions.

**Table 3:** Opinions about impact of restriction on medical education, if any, according to students and deans

Impact on Medical Education	Students		Deans				
	*M*	*SD*	*M*	*SD*	*t*	*p*	*d*
Student involvement in patient care	1.88	.91	1.88	1.20	−.00	1.00	0.00
Assessment of competences	1.96	.98	2.00	1.25	−.21	.83	−0.04
Feel part of the team	1.89	.97	1.83	1.24	.26	.80	0.06
Development of communication written skills	1.76	1.03	1.88	1.26	−.54	.59	−0.12
Groundwork to be a MD	1.76	1.06	1.83	1.34	−.32	.75	−0.07

Answered on a 5-point Likert scale (from 1 = very negative to 5 = very positive)

Regarding the consequences for patient care, both deans and students thought that restricting students to write in EMR has a negative impact on patient care (*M*_*deans*_ =1.96, *SD* = 0.94, *M*_*students*_ = 2.46, *SD* = 1.11). Deans considered the impact more negative than students did, *t* = 2.60, *p* < .02, *d* = 0.45. Students and deans thought that restricting documentation by students in MR negatively impacts aspects of patient care regarding risk of medical errors, opportunity of malpractice suits, use of resident’s or physician’s time, opportunity to find information on MR, completeness of patient information, and care team’s understanding of patient problems. The consequences on care team’s understanding of patient problems, was neutral for deans and more important for students (*t* = -2.00, *p* < .05, *d* = .41) (Table 4).

**Table 4:** Opinions about impact of restriction of student documentation on MR on patient care, if any, according to students and deans

Impact on Patient Care	Students		Deans		*t*	*p*	*d*
	*M*	*SD*	*M*	*SD*			
Possibility of medical errors	2.30	1.03	2.56	1.36	−1.19	.23	0.25
Opportunity of malpractice suits	2.30	1.06	2.32	1.11	−.10	.92	0.02
Use of resident’s/physician’s time	2.68	1.12	2.84	1.25	−.68	.48	0.14
Opportunity to find information on chart	2.56	1.12	2.96	1.21	−1.73	.84	0.36
Completeness of patient information	2.54	1.13	2.80	1.26	−1.10	.27	0.23
Care team’s understanding of patient problems	2.46	1.12	2.92	1.15	−2.00	< .05	0.41

Answered on a 5-point Likert scale (from 1 = strongly agree to 5 = strongly disagree)

On a list of topics that may justify the restriction to write students’ notes on EMR the concern most identified was medical liability, as indicated by 85% of both students and deans. The need for co-signature was more important for deans (mentioned by 73% of them) than for students (47%, *χ* ^*2*^ = 6.89, *p* = .01). Billing aspect was a concern for 69% of deans and only for 27% of students (χ2 = 21.23, *p* < .00). Placement of incorrect information on EMR (deans: 73%, students: 57%), and accreditation issues (deans: 65%, students: 46%) were perceived as equally important by both deans and students.

The existence of a mechanism to assure the co-signature of students’ progress notes in EMR was known by 75% of the students and 77% of deans, while 6% of students and 8% of deans did not know about its existence (*χ2* = 0.25, *p* = .88).

## Discussion

This survey including data from two important stakeholders in medical education - deans and students from medical schools - provides important information on perceptions and concerns about use of EMR by students. This aspect of medical education needs more understanding, especially because worldwide ever more university hospitals are using EMR at inpatient and outpatient services. Results indicating differences in perceptions between deans and students raise questions about the relationship between the planned curriculum, which deans are most aware of, and the real clinical learning environment, which is what students experience in daily practice. We found differences and similarities between perceptions of deans and students, which indicate directions for further improvement of the incorporation of the use of EMR in medical education.

Only about half of the deans and students report the existence of a formal learning program to teach how to write and assess progress notes in EMR and the existence of a policy regarding the placement of student notes in EMR. For students and curriculum planners an explicit and well-known policy about this subject would be beneficial. School authorities have to look for solutions to overcome the educational deficit of not having the use of EMR by students in their medical curriculum. Students more often found ways that allowed them to use EMR than assumed by deans. With the increase in the use of EMR at university hospitals, the findings that students perceive less possibility of documenting in the EMR compared to paper-based MR suggest a deterioration in the quality of the learning environment. Our results show that deans are not aware of this difference in student access to medical records depending on its format.

These results call our attention about these disagreements; a useful learning experience must align what planners and learners expect from the experience (participate and write notes in MRs), with the possibilities to do it in real life situations. If these differences between the expected or desired and the real situation are not worked out, learning experience quality will reduce. In their presentation of experience-based learning Dornan [21] concluded that a decrease of student participation in clinical practice may have a deleterious effect on learning. Designing learning environments has to be done with the participation of students, teachers and designers [19] The findings of the current study show discrepancies between the designers of learning experiences and the perception of students. A clinical learning environment is a complex setting that deserve in depth studies, with the participation of all actors involved, to understand the manner students find their way to learn while they are authorized to be “doctors” [22]. There are non-curricular resources available in the clinical environment to acquire the required competencies to write on MR. Usefulness of these opportunities was perceived differently from curriculum planners and final users. Most of the deans assumed that observing and imitating attending physicians were resources that students may use to obtain the necessary skills to manage (E)MR. Only one out of three students saw this option as a learning opportunity. These results again raise questions about discrepancies between the planned learning activities and the real situation.

Restriction of students to place notes in patient EMR, may have negative impact on medical education and patient care, according to both deans and students. These findings remark the importance of finding a solution for this urgent educational problem. Restriction of students’ use of EMR causes students to not feeling part of the team of care, not having the possibility to write findings on patient’ charts, and having obstacles to learn and being assessed on writing communication skills. Student’s participation may enhance the amount and quality of relevant information about some aspects of patient conditions and, therefore, limiting their participation may decrease care team understanding of patient’s concerns. These results stress the need to find opportunities to improve quality of education and patient care, avoiding the tacit exclusion of the student in the team of care, as a method to develop real tasks in the design of the learning experiences in clerkships [23]. Furthermore, concerns about economical or legal consequences when students are allowed to put notes in EMR should be shared between different stakeholders in order to align interest and decrease negative effects.

The development of EMR has been guided mainly for administrative purposes, but now seem to block that the students are involved in real clinical tasks. From the point of view of instructional design [24] and looking for the construction of powerful learning environments [19, 21], we need students acting and feeling as doctors, part of the team of care; we need that educators, teachers and students participate together in the construction of real life learning tasks. The hidden curriculum and the non-curricular opportunities to learn found in this study need to be clarified and incorporated in the curriculum. The opportunity to work with EMR developers to introduce educational tools, like the RIME proposal [25] and other tools that enable students to use EMR without resign their role as a part of the clinical team, needs to be seriously addressed. The integration between EMR and medical education had not been accomplished [7, 10]. Faculty development in medical bioinformatics and definition of specific competences in informatics for health professionals are necessary steps to take advantage of the prospective applications of EMR and e-health on medical education [10, 15, 26].

The results of our study, which is the first one that compares the perceptions of deans and students on this issue, contributes to understanding the complex situation of the use of a relatively new technological tool in a very traditional learning site as the clinical ward. A limitation of the study is that survey studies are susceptible for social desirability in answering. Our findings resonate with findings of earlier studies, for example a survey with clerk directors at USA, which has shown that medical students use EMR at higher rates than physicians at practice, but once again rises concerns about the link of practice with curricular design [27]. From our results it is clear that a comprehensive approach with the participation of all actors is necessary to fully understand this complex situation. A further study focusing on the amount and type of supervision needed for medical students interfacing with the EMR is suggested. Educators, teachers and students point to different issues and consequences of the use of EMR in medical education, which impact the student-teacher, student-patient and patient-teacher relationships.

## Conclusions

The spreading of the use of EMR at university hospitals with restricted access to students and the lack of regulatory policies and devoted learning activities in this context may be hampering the acquisition of clinical competencies for students. This study underlines that the role of the student during clinical clerkships needs to be redefined to resolve the current interference between use of EMR systems in hospitals and the quality of medical education for students. There is a need for a clear policy and educational program on the use of EMR by students. Deans and students, in our study and in other published studies and position statements [5, 8, 15, 17, 28] are convinced about the added value of students being able to write in EMR both for the quality of patient care and education. The Alliance for Clinical Education, an organization of clinical educators, has published critical principles of the use of EMR for medical studies, including that students must document their findings on EMR, while reviewed for content and format – which requires appropriate supervision - and schools must develop a set of medical student competencies related to charting in the EMR [8]. Concerns about medical liability, billing, placement of incorrect information on EMR, and accreditation issues have to be resolved in a constructive collaboration between the involved stakeholders, including also EMR designers and hospital administrators. The restricted students’ use of EMR demonstrates that medical accountability is top priority, with suffering quality of medical education as its consequence. A better balance between both major aims is required.

In order to create a powerful work-placed learning experience the design of learning tasks during clerkships should define an active and well-defined participation of students in the different aspect of patient care, including the use of EMR. We recommend intense supervision of students’ work in EMR. The opportunities to take advantage of the technological advances of EMR and e-health is likely to increase with the coordinated participation of the different actors at the modern clinical site.
